# Kill and Clearance in HCC: An Approach Based on NK Cells and Macrophages

**DOI:** 10.3389/fonc.2021.693076

**Published:** 2021-09-07

**Authors:** Maryam Mehrabi, Fatemeh Amini, Shima Mehrabi

**Affiliations:** ^1^Independent Researcher, Newcastle, United Kingdom; ^2^Vitaccess, Oxford, United Kingdom; ^3^Internal Medicine, Iran University of Medical Sciences, Tehran, Iran

**Keywords:** NK, macrophage, monocyte, myeloid-derived suppressor cell, hypoxia, phagocytosis, liver homeostasis, hepatocellular carcinoma

## Introduction

In our previous perspective, we investigated the role of hypoxic lesions in the development of necrotic zone in solid tumors (1). We suggested the increased infiltration of macrophages/monocytes to the pre-necrotic zone and under the influence of chemoattractants (VEGF, CCL2, and CCL5), danger signals, and a trail of necrotic debris as a mechanism to maximize their opportunity to clear the lesion. They continuously sample and mediates recognition of damage-associated molecular patterns (DAMPs), including heat shock proteins, cytokines, DNA, RNA, metabolic ATP, HMGB1, histones, altered carbohydrate, and negatively exposed phosphatidylserine (PS) on the surface of necrotic corpses or apoptotic cells. We suggested gradual alteration of gene expression in macrophages eventually turns off their phagocytic machinery leaving uncleared cell corpses as a rich source of building blocks for cancer stem cells. Even subtle differences in the internalized ligands could have far-reaching consequences on cytokine production and antigen presentation to NK cells.

Here, we propose a synergistic act of kill and clearance by NK cells and macrophages as a therapeutic strategy in HCC. We have provided a comprehensive mechanism underlying human NK cell and macrophage dysfunction in HCC, investigated the crosstalk between NK cells and a group of myeloid-derived suppressor cells (MDSCs) with a typical monocytic phenotype (CD14+ HLA-DR-/low), and the immunosuppressive role of hypoxia in HCC. We have also outlined the possible future research directions to target the crosstalk between NK cells and macrophage/MDSC to enhance kill and clearance by NK cells and macrophages in HCC and other solid tumors.

## Immunology of Liver During Homeostasis and HCC

**Figure 1:**
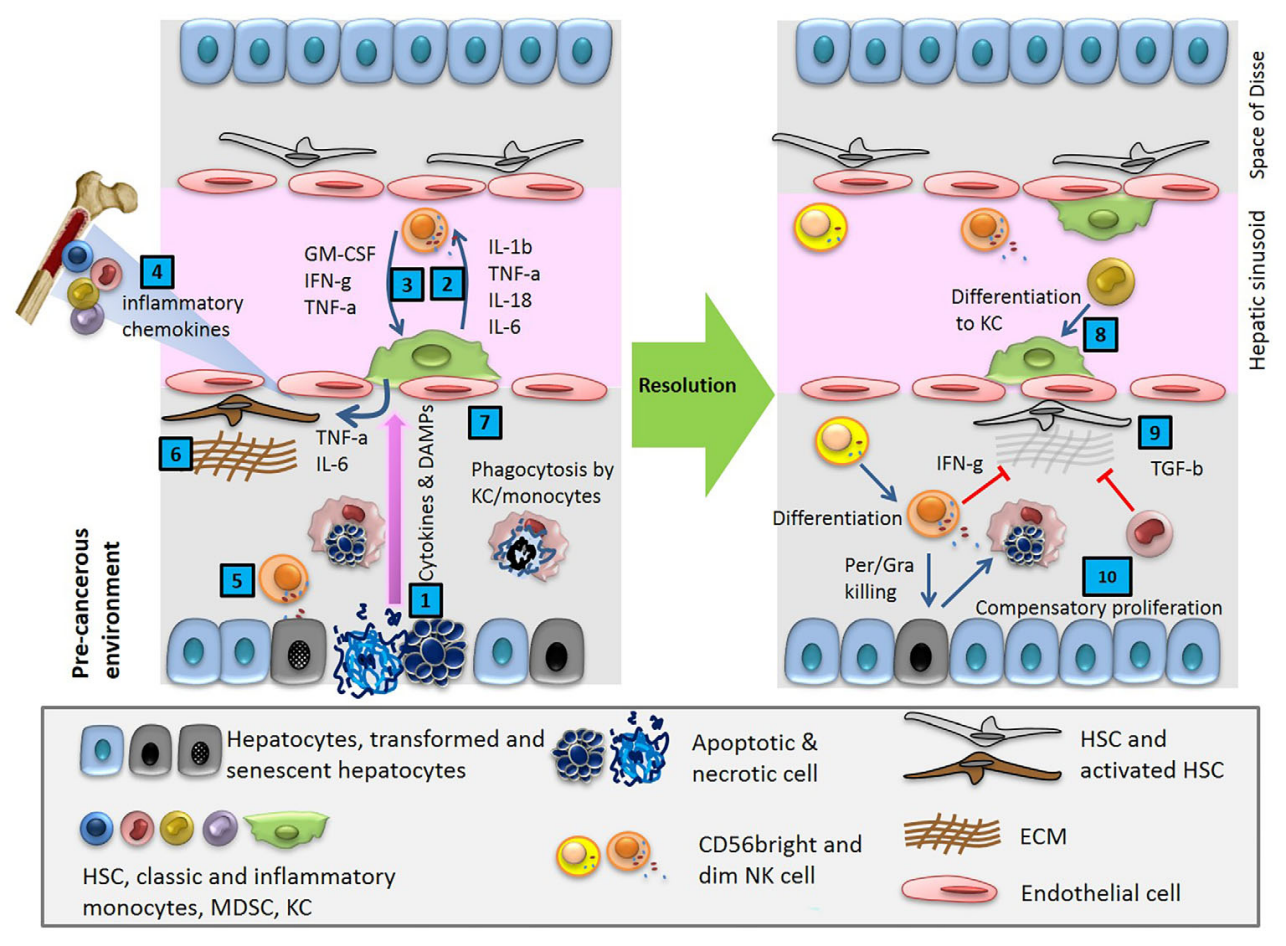
Cytokines and DAMPs released in pre-cancerous environment activate innate immune cells, resident macrophages (KC) and NK cells, and induces HSCs trafficking from BM. After a cycle of cytotoxic NK cell mediated cell death and phagocytosis of debris by macrophages, the tissue reaches homeostasis.

The adult human liver receives a mixture of 75% deoxygenated blood from the portal vein and 25% oxygen-rich blood from the aorta. The former contains digested nutrients from the entire gastrointestinal (GI) tract, the spleen, and the pancreas. During homeostasis, the liver is constantly bombarded with gut-derived products in a strictly regulated microenvironment, which removes dangerous stimuli and resolves inflammation. This organ maintains a balance between pro-inflammatory (e.g. TNF-α, IL-1β, IL-2, IL-12, IL-15, and IFNγ) and anti-inflammatory (e.g. IL-10, IL-13, and TGFβ) cytokines in the absence of pathological inflammation or infection (**Figure 1**) (2). Production of these cytokines through normal physiological processes is controlled by a network of hepatic lymphoid, myeloid, and non-hematopoietic cell populations (3). With enormous regeneration capacity, differentiated hepatocytes can enter the cell cycle and repair the injured tissue after acute damage (**Figure 1**) (4, 5). Failure to clear dangerous stimuli due to any dysregulated inflammation could drive chronic infection and pathology (**Figure 3**).

Tumorigenesis is generally believed to be the consequence of accumulated mutations. Genetic changes in mature hepatocytes are a common condition caused by multifactorial processes such as metabolic liver disease, and alcoholic or non-alcoholic liver disease (6, 7). Despite variable causes, primary liver cancers arise almost exclusively in the setting of chronic inflammation, in which stress stimuli within hepatocytes initiate the death of liver cells, the production of DAMPs, and the influx of activated immune cells (8, 9). The sequence of chronic necroinflammation and release of DAMPs during tumor growth results in the upregulation of a number of immune regulatory pathways aimed at compensatory liver regeneration. Meanwhile, the cycle of orchestrated events including expansion and accumulation of tumor-promoting immunoregulatory cell populations like myeloid-derived suppressor cells (MDSCs), epigenetic and metabolic changes resulting in immune cell tolerance, induction of negative regulators of pro-inflammatory signaling pathways, and development of T-cell exhaustion results into the induction of liver fibrosis and/or subsequent cirrhosis, which often precedes hepatocarcinogenesis.

Recent Genomic profiling has provided an overview of significant drivers of HCC (10–12) with about 40 somatic alterations in coding regions, including the genes encoding the tumor suppressor p53 and cell-cycle regulators, as well as molecules involved in WNT-β-catenin signaling, epigenetic modification, oxidative-stress pathways, the RAS–RAF–MAPK, and PI(3)K–AKT–mTOR pathways (13). The frequency of these mutations depends on underlying risk factors. Despite all the development in the molecular characterization of HCC, little progress was achieved in stratifying patients and developing predictive biomarkers for targeted therapies.

In search of new therapeutic strategies, an extensive immunogenomic analysis of diverse cancer types are published in “The immune landscape of cancer”. Using TCGA molecular platforms and Hematoxylin-Eosin (H&E) staining data analysis of 10,000 clinical samples including HCC, the research predicts intrinsic and extrinsic regulators of tumors with a critical role in the leukocyte population and infiltration in the TME (14). The collective intrinsic data suggest the influence of seven immune-related transcription factors, including interferon and STAT-family. The extracellular networks imply direct cell-to-cell contact between immune cells or communication *via* soluble molecules. Proinflammatory cytokines, such as IL-6 and TNF, which activate the transcription factors STAT3 and nuclear factor-κB (NF-κB), respectively, have been reported to be important for the development and progression of HCC. Hepatocyte-specific inhibition of STAT3 has been shown to inhibit tumor growth in a mouse model of chemically induced HCC (15). The role of key receptors and ligands, such as transforming growth factor-β1 (TGFβ1), CXCL10, CXCR3, and receptor-ligand pairs, such as the CCL2-CCR2 axis, is highlighted in the cellular communication network. An important key point manifested in the immune subtypes is that the immune system context can dictate the immune cell interactions. Interestingly, a dramatic increase in the number of macrophages and the rarity of NK cells is an obvious trend in all the HCC studies.

## Macrophages and NK cells in healthy liver

The liver is a tightly controlled immunological network with one of the largest populations of innate immune cells. Additionally, intrahepatic lymphocytes are often present in the sinusoidal lumen. Deregulation of this network is a hallmark of chronic liver disease and HCC. As key components of the innate immune system, resident macrophages known as Kupffer cells (KC) and NK cells play vital roles in maintaining organ homeostasis and rapid response to potentially dangerous stimuli. They continuously survey their microenvironment for danger and are ready to respond by entering an activated state characterized by cytokine secretion, phagocytosis, and, on occasions, direct cytotoxicity (**Figures 1** and **3**) (16).

### Resident Macrophages (Kupffer Cells)

The liver sinusoid has a lining of discontinuous endothelium and KCs, allowing for the removal and degradation of immunogenic molecules in the liver (17). Resident macrophages, KCs, constitute a third of the liver non-parenchymal cells and located within the hepatic sinusoids, in close contact with both the sinusoidal endothelium and hepatocytes. Having the largest population of resident macrophages, the liver has 80–90% of total resident macrophages present in the body. They are capable of responding to cytokines, Toll-like receptor (TLR), RIG-like receptor, and NOD-like receptor signaling, and express a variety of receptors, including the scavenger receptor cysteine-rich (SRCR) superfamily members (18). KCs are equipped with a massive array of PPRs, complement receptors, and Fc receptors, through which they respond with increased phagocytic activity and production of inflammatory cytokines. The liver microenvironment alters the physiologic function, population density, and cytologic characteristics of KCs. For example, periportal KCs at the first point of contact for incoming blood have a greater phagocytic capacity than smaller KCs from midzonal and perivenous regions (19, 20). KCs play a central role in the liver systemic immune responses, have essential roles in immune regulation, tissue repair, and liver regeneration. In liver disease, they have a phagocytic function and release cytokines such as TNFα and IL-6, both important mediators in hepatic inflammation and fibrogenesis. There is cumulative evidence from murine transplantation experiments that KCs can derive from the bone marrow (BM) monocytes (21–24). Monocytes in tissue also participate in tissue healing, clearance of pathogens and dead cells, and initiation of adaptive immunity.

### Hepatic NK cells

Human NK cells are derived from BM and phenotypically defined as CD3^−^CD56^+^ large granular lymphocyte. They are part of the innate immune system and a key player in cancer immune surveillance. NK cells form up to 50% of the intrahepatic lymphocytes, approximately 5 times higher than the proportion in peripheral blood. Hepatic NK cells are constitutively activated, displaying significantly higher cytotoxic activity against tumor cells compared with circulating NK cells (3, 25). Two subsets of NK lymphocytes, CD56bright and CD56dim, with different functional outcomes contribute to the liver NK cells. Cytolytic activity is mostly confined to the CD56dim subset, whereas cytokine production is generally assigned to CD56bright cells. Hepatic NK cells can control the progression of HCC *via* two mechanisms, killing tumor cells and targeting liver fibrosis.

NK cells express a wide array of germline-encoded inhibitory and activating receptors in a stochastic pattern that bind and detect various tumor ligands, contrary to T and B cells which require somatic gene rearrangements to generate receptor diversity and specificity. They are involved in nonspecific, intrahepatic cell killing due to downregulation of the major histocompatibility complex class I (MHC-I) on tumor cells. The MHC-I downregulation leads to loss of the NK cell inhibitory signal and activation of killing pathways (26). Recognition of DNA damage in cancer cells by the activatory receptor, NKG2D, is another detection pathway (27), while some others remain unknown. NK cells exert antitumor effects by exocytosis of perforin/granzyme-containing granules, induction of apoptosis in target cells, and production of various cytokines that augment the functions of other immune cells (28, 29), and can change the course of tumor development (30–32).

Hepatic NK cells can also control the progression of HCC *via* anti-fibrotic properties. Liver fibrosis is characterized by the differentiation of hepatic stellate cells to myofibroblast and excessive deposition of extracellular matrix proteins in the liver. It is a common scarring response to various forms of chronic liver disease and is associated with excess hepatocellular death (33). The anti-fibrotic property of NK cells through targeting activated hepatic stellate cells is a major pathway they provide immunosurveillance to HCC (34–37). However, this property is often suppressed in human HCC. There are several possible explanations for this, including STAT3-mediated upregulation of immunosuppressive cytokines IL-10 and TGFβ (38), phagocytosis of NK cells by hepatic stellate cells (39), NK cell exhaustion, and the dysregulation of NK cell-activating ligands (40).

HCC is associated with a dramatic decrease in the frequency of circulating and intrahepatic NK cells as well as their impairment in cytotoxicity and cytokine production (41, 42). This is mainly reflected in the reduction of CD56dim NK cell subset rather than CD56bright and tends to decrease further in advanced-stage HCC patients (43, 44). It has been shown that low intratumoral NK cells among tumor-infiltrating lymphocytes (TILs) have shorter disease-free survival (DFS) and overall survival (OS) and restoration of NK cell activity after curative surgery in clinical trials is associated with recurrence-free survival (45).

## Trafficking and Accumulation of Monocytic Cells in HCC

### Monocyte Trafficking in HCC

**Figure 2:**
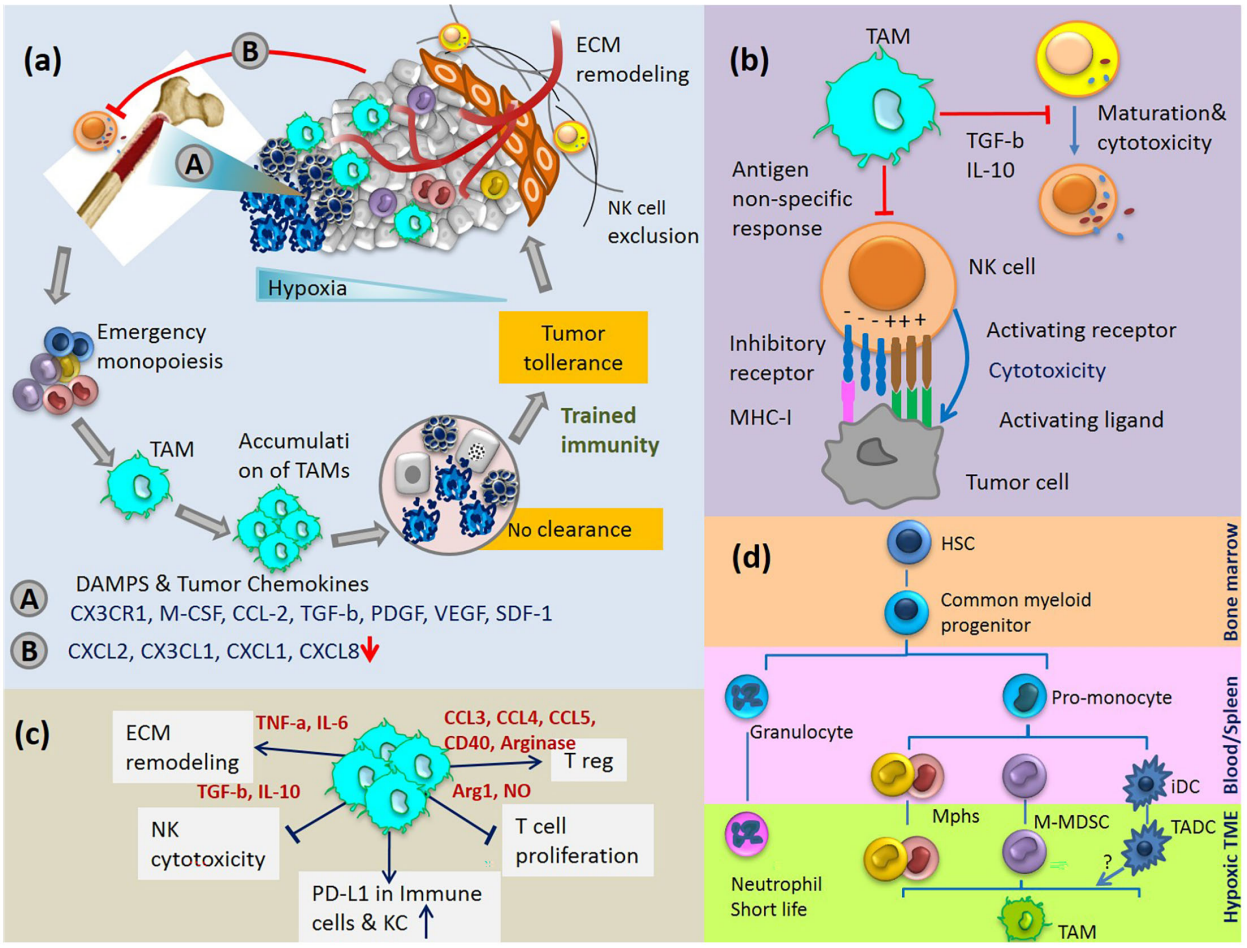
**(A)** Cycle of emergency monopoiesis, accumulation of immature suppressive macrophages in TME, inhibition of CD56dim NK cell egress from BM and their exclusion from tumor are part of the synchronize program which causes immune tolerance to HCC. **(B)** TAM inhibits NK cell cytotoxicity and maturation. **(C)** Immunosuppressive activities of TAMs. **(D)** TAMs differentiation pathway: myeloid cells originate from HSCs in the BM and differentiate into various myeloid cell lineages in blood, spleen and tumor tissue. Various monocytic cells in TME change to polarized phenotypes between M1-M2. iDC, immature dendritic cells; TADC, tumor-associated dendritic cells.

Molecular alterations of hepatic gene expression mediate microenvironment changes that favour tumour cell colonization. The tumour-derived secreted factors in an inflammatory microenvironment induce rapid infiltration of BM progenitor cells to the liver and dictate their differentiation programs. For example, stress stimuli in TME upregulate the gene expression of hepatic SDF-1 localised to the biliary epithelium. SDF-1 is a potent HSC chemoattractant, and plays a key role in their migration from BM to circulation and eventually homing to the site of insult in a concentration-dependent manner (46). Gene expression of SDF-1 is directly regulated by the transcription factor HIF-1, resulting in selective surface expression of SDF-1 protein in direct proportion to reduced oxygen tension. As a result, migration and homing of circulating CXCR4-positive progenitor cells increase to the hypoxic area of tissue. It has been shown that blockade of SDF-1 or CXCR4 on circulating cells prevents progenitor cell recruitment to the sites of inflammation (47). The recruited BM cells play an important role in the generation of fibrosis and tumor progression. In a complex coordinated process, these cells together with liver cell compartments, express a specific set of growth factors and cytokines, which allows the immune escape of tumor cells in an immune-suppressive environment (**Figure 2A**).

During homeostasis, monocyte trafficking from BM is under the control of chemoattractants, such as IL-3, stem cell factor (SCF), and macrophage colony-stimulating factor (M-CSF). Monocytes stay in circulation for about 3 days; then randomly leave to tissues, where they play a key role in clearance and phagocytosis. There is also a continuous loss of Kupffer cells in the healthy liver, which is balanced by the formation of new macrophages from circulating monocytes and to a small extent from the self-renewal of existing KCs (**Figure 1**). The network of cells concerned with macrophage production in the liver is part of the mononuclear phagocyte system.

Emergency hematopoiesis during liver disease is characterized by a shift in cell fate resulting in higher production of particular immune cells at the expense of others, as such increased production of monocytes compared to lymphocytes (48, 49). The half-life of BM-derived monocytes in this condition temporarily reduces to 12 h. They contribute to immune defence by direct recruitment to the injured tissue and subsequent entry into lymph nodes *via* afferent lymphatic channels (50, 51). This process is accompanied by long-lasting effects and results in an altered activation state and increased number of monocytes even weeks after inflammation, a phenomenon called ‘‘trained immunity’’ (52). It has been proposed that cytokines, like IFNγ and IL-1β, secreted in BM by immune or non-immune cells are crucial for these long-lasting training effects (53). There is no doubt that these cells are ‘‘trained’’ through epigenetic changes since HSCs isolated from animals injected with β-glucan preserved their capacity to generate more myeloid cells 12 weeks post-transfer into untrained animals (54).

Understanding the relationship between progenitor cell products derived from BM and monocyte-to-macrophages differentiation is of great interest (55). Recent studies in human and transgenic mouse models suggest that different subsets of macrophages in tissue originate from a distinct population of monocytes in peripheral blood (21–24). The main monocyte-associated antigen marker is CD14, the LPS receptor, being the most specific (56). During maturation, human monocytes gain a progressive increase in CD14, CD11b, CD13, CD36, and CD45 expression, with a mild decrease in HLA-DR and CD15. Mature monocytes show expression of bright CD14, CD33, CD36, CD38, CD64, variably bright CD13, and low CD15 (57, 58). Another marker antigen, CD68, is broadly expressed in all mononuclear phagocytes and related to the lysosome-associated membrane protein (LAMP) family. However, its function is still obscure. Apart from marker antigens, levels of chemokine receptors for fractalkine (CX3CR1) and CCR2 (MCP1) seem to distinguish monocyte subsets that give rise to inflammatory and resident macrophages. The majority of the cells migrate to the site of inflammation under the influence of Soluble mediators including vascular endothelial growth factor (VEGF), platelet-derived growth factor (PDGF), transformation growth factor-β (TGFβ), chemokine (C-C motif) ligand (CCL2), and macrophage colony-stimulating factor (M-CSF). The expression of glypican-3 (GPC-3) on the surface of HCC cells may also promote monocyte recruitment (59). The rationale is that distinct subsets of monocytes are recruited at different stages of liver injury and tumor progression, with the first wave of monocytes facilitating the removal of dead hepatocytes and debris and the later phase promoting the resolution of inflammation and tissue repair (50, 60).

In the context of HCC metastasis, increased level of M-CSF and extensive infiltration of macrophages have been associated with intrahepatic metastasis and recurrence (61). Moreover, pharmacological approaches to directly target TAMs, *via* knocking out CCL2 or other TAM-specific chemokines, reduced migration and invasion of HCC cell lines (17).

### Myeloid-Derived Suppressor Cells in HCC

In patients with HCC, a group of myeloid-derived suppressor cells has been identified based on the expression of CD14 low level of HLA-DR, with a typical monocytic phenotype. The number and percentage of these cells increases in blood and the site of insult as time passing after tumor inoculation (62). They can induce liver damage and hepatocyte apoptosis. These cells also contribute significantly to the immunosuppressive microenvironment of tumors using multiple mechanisms, including suppression of T cell proliferation, generation of induced regulatory T cells, and upregulation of inhibitory surface protein, PD-L1, in KC. They repress the antitumor activity of NK cells by inhibiting perforin-dependent cytotoxicity.

It appears that the migration of MDSC to the site of the tumor is governed by the same mechanisms as monocytes and neutrophils. Currently, there is no clear evidence that MDSCs may have a unique pattern of migration distinct from their control counterparts (63). However, this issue needs to be formally investigated.

MDSCs are present in the healthy liver and expand during chronic liver disease (64). The expansion of MDSCs is well known for acute bacterial and viral infection as well as other stress stimuli. However, they quickly differentiate to mature macrophages, neutrophils, or DCs, rather than accumulating in the site of insult. This phenomenon is only common in cancer where MDSCs maturation is affected by multiple unknown factors produced by tumor and tumor stroma. The extended stimulation of MDSCs by various growth factors and cytokines in TME blocks their terminal differentiation and they expand variably to immature phenotypes of TAMs consistent with tumor heterogeneity (**Figures 2A, D**) (65–67).

## Trafficking and Differentiation of NK Cells in HCC

### NK Cell Trafficking in HCC

HCC is associated with a dramatic decrease in the number of NK cells and impairment in their cytotoxicity. This is partly dictated by the chemokine milieu in TME, which preferably attracts CD56bright subset of NK cells rather than CD56dim. Moreover, reduced expression of CXCL2, CX3CL1, CXCL1, and CXCL8 results in a reduced number of CD56dim subsets in TME (68). Another important driving factor for these changes is TGFβ1 in TME. TGFβ1-mediated increase of the miR-23a-27a-24-2 cluster in NK cells induces downregulation of the surface expression of CX_3_CR1 (69). CX3CR1 is a receptor to CX3CL1 (fractalkine) and one of the main regulators of NK cell egress from BM and extravasation to the site of tissue. This ligand is selectively expressed by CD56dim NK cells (70). Moreover, the miR-23a-27a-24-2 cluster hampers NK cell cytotoxicity through downregulation of perforin/granzyme B expression. Interestingly, TGFβ1 is also responsible for upregulation of the mentioned microRNA axis in hepatocellular carcinoma cells (71), as well as in CD8 T cells. Given that this is not limited to HCC and has been reported in several tumors, makes the miR-23a-27a-24-2 axis a potential target for the treatment of HCC (72).

### NK Cell Differentiation in HCC

Besides receiving myeloid and lymphoid cells from BM, the adult human liver contains populations of HSCs, which form colonies upon *in vitro* culture. These cells may contribute to the local HSC differentiation under stress (73, 74). Lymphocytes extracted from the adult human liver contain a high level of recombinase-activated genes 1 and 2 (RAG1 and RAG2). These enzymes are involved in lymphocyte gene rearrangement and recombination, suggesting lymphocyte differentiation can occur in the adult liver (75). Murine models of liver transplant also well describe this phenomenon (19, 76), and c-kit+ stem cells in adult mice liver have the potential to reconstitute multiple lineages and BM stem cells (76–78).

Complete maturation of NK cells in HCC is accompanied by the chemokine receptor repertoire, which would define their re-circulation toward the site of insult. There is a substantial number of NK cells in afferent lymphatic vessels. This is like naïve T cells and supports the hypothesis that tumor lymphatic organ is the site for NK cell maturation, acquisition of cytotoxic properties, and tolerance to self (KIR expression). Alternatively, NK cells could mature to effector NK cells during the local immune response in the malignant tissue (79).

### MDSCs and NK cells

*In vitro* culture of NK cells, isolated from blood or tumor in human HCC, with autologous M-MDSCs (CD14^+^ HLA-DR^-/low^) significantly reduces the cytotoxicity and release of IFNγ in NK cells. This phenomenon in the liver, MDSC-mediated NK cell impairment, is a general mechanism in all types of liver disease, but the frequency and accumulation of MDSCs in blood and the site of insult happens only in cancer (80).

Several *in-vitro*, clinical, and preclinical studies have investigated the pathways to reduce MDSC accumulation in TME and the consequences on NK cell cytotoxicity (81). As a valuable source of data, these cases shed light on the future therapeutic strategies for kill and clearance based on NK cells and macrophages. A few notable examples are as follow:

The mechanism of MDSC accumulation in HCC is shown to be activated by direct contact between NKp30, the NK-activating receptor, and unknown ligands/factors on MDSC. This inhibitory process causes a significant reduction in surface expression of NKp30; but is not dependent on NKG2D, CD16, CD94, NKp44, CD69, and NKp80. Also, blocking arginase activity or the production of NO in MDSC does not affect this process (62). Blocking NKP30 interaction with its counterparts on MDSC can inhibit the trafficking of MDSC to the site of tumor, reduce the NO production by MDSC, and results in the enhancement of NK cell cytotoxicity in HCC.

Blocking MDSC chemoattractants, such as CCL2 and CCR5, is another strategy to reduce MDSC migration and homing to TME. As an example, inhibition of CCL2 produced by MDSCs, inhibits JAK/STAT3 pathway and reduces MDSC trafficking to the site of tumor (82), while targeting STAT3 in tumor-bearing mice, exhibits a higher expression of the NK activation markers NKG2D, CD69, Fas ligand (FasL), granzyme B, perforin, and IFNγ, resulting in reduced tumor growth (38, 83).

Another approach, the improvement of MDSC differentiation using vitamin A, D, and E in clinical trials, provided promising results (84). While vitamin D3 decreased the level of immature MDSCs, patients with vitamin D deficiency had lower NK-mediated cytotoxicity (85). Vitamin E treatment also can target this cross-talk, enhances immune responses through decreasing NO production by MDSCs (86), and it is known that MDSCs impair NK cell function *via* the production of NO (87). Thus, inhibition of NO production offers a strategy for targeting MDSC-NK crosstalk and their role in the kill and clearance of solid tumors.

## The Role of Hypoxia in Tumor Progression

The hypoxic microenvironment in advanced solid tumors is the result of tumor cell outgrowth and imbalanced vascularization (88). This phenomenon plays a critical role in metabolism, phenotypic regulation of the cancer stem cells, and epithelial-mesenchymal transition (EMT) (89). Responses to hypoxia are nearly instant and include short-term changes in the activity of biomolecules to long-term adaptations of global gene-expression programs at the cellular, tissue, and organismal level. Strong nuclear accumulation of the transcription factor HIF-1α at the cellular level modulates the expression of nearly 1000 genes involved in hypoxic adaptations, blood vessel formation, cell differentiation, and inflammation. In HCC, the high expression level of HIF-1α strongly correlates with the adverse prognosis and progressive stages of the tumor. It has been established that HIF proteins activate specific signaling pathways, such as Hedgehog and Wnt/β-catenin, which accelerate the EMT and aggressive capacity of HCC cells (90, 91). Hypoxia also leads to the accumulation of intracellular reactive oxygen species (ROS), which induces GLI1-dependent EMT progress (90, 91). Conversely, developed HIF inhibitors show immense promise for further development of anti-cancer drugs (92).

Hypoxic effects are not limited to hepatocytes and alter immune cell functions by metabolic reprogramming and switching from oxidative phosphorylation to aerobic glycolysis. This metabolic switch in macrophages is essential for the production of proinflammatory mediators like IL1β and causes activation of HIF-1α (93). However, such influence on liver KC function is presently unknown. Cells in healthy liver receive 75% of blood from the portal vein and are in a constant state of low oxygen tension (94). The fact a hypoxic response is not induced during homeostasis suggests that cells in the liver need a higher threshold of oxygen deficiency to respond or they may have a unique pattern of response to hypoxia.

Recent studies from several groups implicate hypoxia as a very important mechanism that converts monocytes, myeloid cells, macrophages, and MDSC into much more potent immune suppressive cells (**Figure 2D**). These cells differentiate into tumor-associated macrophages quite quickly with profound immunosuppressive activity (95).

Macrophage activation *in vitro* is typically classified as two polarized states, M1 and M2 (96). However, TAMs are phenotypically and functionally placed in a spectrum between M1 and M2, rather than fitting one of them (97, 98). Polarization of TAMs reflects differential changes in TME (99). Changes including oxygen tension and production of lactic acid inhibit glycolysis and derive TAMs metabolic changes towards oxidative phosphorylation (OXPHOS) (100). Upregulation of HIF-1 in hypoxic condition induces production of NO by TAMs, which further promote genetic instability and malignant transformation of cancer cells (100). This master transcription factor is regulated by NF-κB and induces profound changes in the expression level of angiogenesis- and metastasis-related genes, such as VEGF, FGF2, MMP7, and MMP9 (101, 102). Consequent to this is the recruitment of more macrophages, and release of pro-inflammatory cytokines, such as TNFα, IL-1β, MIF, CCL3, and COX2, as well as M2 markers, such as IL-10 and arginase 1. The continued upregulation of HIF is part of the regenerative and immunosuppressive response (103).

Hypoxic alterations result in more potent nonspecific immunosuppressive activity of monocyte/macrophages inside the tumor compare to lymphoid organs. HIF-1 directly binds to a transcriptionally active hypoxia response element in the PD-L1 promoter and increases PD-L1 expression in MDSCs, TAMs, DCs, and tumor cells (104). MDSC lacking HIF-1 did not differentiate into TAM within the TME but instead acquired markers of DC (105, 106).

Hypoxia causes an increase in CD45 tyrosine phosphatase activity of monocytes and MDSCs, which results in the selective decrease of STAT3 activity in the hypoxic region of the tumor. The upregulation of CD45 phosphatase activity is mediated by the disruption of CD45 protein dimerization caused by increased sialylation of CD45. Treatment with sialidase abrogates the effect of hypoxia on STAT3 activity and differentiation of MDSCs to TAM (107). Changes in oxidative phosphorylation and glycolysis in tumors also affect the function of MDSCs. It is now established that activation of myeloid cells leads to a switch towards Warburg metabolism (108).

## Accumulation of Senescent Cells and TAMs in HCC

Transient senescent hepatocytes, at a state of stable cell-cycle arrest, appear frequently in different types of liver disease and pre-cancerous microenvironments. These cells are subject to immune-mediated clearance, which depends on an intact immune response. By contrast, the accumulation of persistent senescent cells at the site of insult is specific to an already established tumor (**Figure 2A**). Secreting chemokines and cytokines like CCL2, these cells induce trafficking and homing of CCR2+ myeloid cells to the liver. In a pre-cancerous environment, the immature myeloid cells differentiate into macrophages and clear the senescent hepatocytes, a process termed ‘‘senescence surveillance’’ (**Figure 1**). However, in an already established tumor, the maturation of myeloid cells to macrophages is blocked by multiple factors and mechanisms, such as tumor-derived growth factors (STAT3, IRF8, C/EBPβ, Notch, adenosine receptors A2B signaling, NLRP3), hypoxia, and negative feedback loops (1, 80). Histopathological samples from HCC patients show a positive correlation between areas of the tumor with senescent hepatocytes (P16+) and myeloid-derived cells (CCR2+). This data suggests the impaired phagocytic machinery of TAMs in HCC which leads to the accumulation of senescent cells. In fact, the secreted factors by senescent hepatocytes suppress liver cancer initiation while accelerating the growth of fully established HCC. To date, many questions remain regarding the specific role of macrophages in the clearance of senescent cells in TME, such as (i) how do macrophages interact with senescent cells, (ii) how does this interaction changes at the different stages of tumor formation and progression, (iii) what is the threshold of senescent cell accumulation and at what threshold of senescent cells accumulation macrophages lose their phagocytic activity? Future comprehensive research is needed to identify other factors linked to senescent accumulation in HCC and their interaction with macrophages.

## Strategies to Enhance NK Cells and Macrophages Kill and Clearance in HCC

To identify the potential targets to unleash the power of NK cells and macrophages in killing the cancer cells and clearing the dead bodies, debris, and senescent cells in TME, it is rational to focus on the key molecular determinants of NK-macrophage crosstalk. In a reciprocal regulation, human macrophages/monocytes produce cytokines which dictate the activation of NK cells, in turn, NK cells orchestrate the macrophage immunity by producing immunomodulatory factors. In the therapeutic strategy based on kill and clearance, the goal is to enhance the phagocytic activity of macrophages and increase number of activated cytotoxic NK cells in HCC (**Figures 2B, C**).

A notable example is the direct interaction between NKP30 and its counterparts on MDSCs, which facilitates the trafficking and accumulation of MDSCs at the site of tumor (62). Blocking this interaction is a potential candidate to boost NK-macrophage kill and clearance in HCC.

The immune checkpoints between macrophages and NK cells may also provide new opportunities to explore the therapeutic effect of their blocking on kill and clearance in TME. As an example, data from patients with B-cell lymphoma suggest that PD-L1/PD-1 signaling on TAMs impairs their phagocytic capacity through SIRPα, an inhibitory receptor expressed mainly by myeloid cells. The data suggest the interruption of the PD-1/PD-L1 axis is sufficient to activate NK cells and enhance cancer cell lysis through degranulation of CD107a (109).

Another strategy to target the crosstalk between macrophages and NK cells is to cut off TAMs replenishment at the site of tumor. Most of these cells migrate from the bone marrow and recruit into TME under the influence of chemoattractants. Therefore, their trafficking is highly dependent on signaling axis like CCL2/CCR2, CCL5/CCR5, CSF-1/CSF-1R and the Soluble mediators including vascular endothelial growth factor (VEGF), platelet-derived growth factor (PDGF), transformation growth factor-β (TGFβ), and macrophage colony-stimulating factor (M-CSF). The expression of glypican-3 (GPC-3) on the surface of HCC cells may also promote monocyte recruitment (59). An implication of this is the possibility to inhibit the establishment of an immunosuppressive pre-metastatic niche *via* TAM suppression and filtration of NK cells *via* blocking these signaling pathways or reducing the soluble mediators (110, 111). Ongoing clinical trials and preclinical models of solid tumors based on this method reported comprehensive advantageous including the increase of NK cells infiltration and improved efficacy of chemotherapy, radiation therapy, and immunotherapy (112). Also, targeting the CSF1–CSF1R pathway improves the efficacy of immune checkpoint inhibitors for the treatment of HCC (CTLA4, PD1) (113), and pharmacological approaches to directly target TAMs, *via* knocking out CCL2 or antibodies and fusion proteins against other TAM-specific chemokines, reduced migration and invasion of HCC cell lines (17).

The new tools like production of human induced pluripotent stem cells (iPSC) derived lymphocytes and genetic engineering give the opportunity to expand the favourite phenotype of immune cells to ex-vivo clinical scale. Bioreactor-derived iPSC-macrophages represent a highly pure population of CD45^+^CD11b^+^CD14^+^CD163^+^ cells with the functional features of professional phagocytes, and iPSC-NK cell offers advantages over the T cell immunotherapy. The Chimeric antigen receptor (CAR) constructs now can provide standardized, targeted off-the-shelf NK cells and macrophages for kill and clearance immunotherapy. As example, researchers have designed CAR-M, which express the intracellular domain of FcRv and promote the phagocytic potential of target antigens. Linking the PI3K p85 subunit to CAR-M-FcRv forms a tandem which has better whole-cell phagocytosis[28]. CAR-NK constructs containing the transmembrane domain of NKG2D, the 2B4 co-stimulatory domain, and the CD3ζ signaling domain offer improved NK cell-mediated killing of tumor cells (114). Nonetheless, critical aspects of cell-based therapies in clinical trials are long-term safety, tolerability, efficacy as well as the tumorigenic potential of the iPSC-derived cell-based treatments.

Several studies implicate hypoxia as a very important mechanism that alters immune cell functions (92). Hypoxia represses NK activation receptors, including NKp46, NKp44, NKp30, and NKG2D, preventing NK cell-mediated cancer cell killing, while enhances the level of MHC class I molecules on cancer cells and their binding to inhibitory receptors on NK cells. In monocytes and myeloid-derived cells, hypoxia causes an increase in CD45 tyrosine phosphatase activity and a decrease in STAT3 activity. Treatment with sialidase abrogates the effect of hypoxia on STAT3 activity and differentiation of MDSCs to TAM (99, 107). MDSCs lacking HIF-1 did not differentiate into TAM within the TME but instead differentiated to DC (105, 106). There has been enormous growing interest in the development of inhibitors to target HIF-1α and the related downstream genes. Therapeutic strategies to disrupting HIF-1α and the related downstream genes would be able to switch TME for kill and clearance by NK cells and macrophages and improve cancer immunotherapies in non-responder HCC patients.

Nanorobots can present potential therapeutic tools for kill and clearance in TME. The recent advances of robotics and nanotechnology can lead to the development of cell-type nanorobots with specific functions. For example, microbivore is a nanorobot designed as artificial phagocyte. It can achieve complete clearance of the most sever septicemic infections in hours or less, far better than the weeks needed for antibiotic-assisted phagocytic defence. Field of nanorobotics can open up new horizons for abundant research which could lead to an amazing range of kill and clearance devices in combination or as substitute for NK cells and macrophages.

## Conclusion

Hepatocellular carcinoma is a complex medical condition associated with a poor prognosis. Symptoms of liver cancer often do not appear until the cancer is at an advanced stage and 70% of patients at diagnosis are ineligible for the curative treatments (resection, liver transplantation, and ablation therapies) (115). The only standard treatment for advanced cancer (sorafenib) also develops long-term drug resistance (116, 117). Therefore, there is a desperate need for new therapeutic strategies in HCC. Spontaneous immune responses against tumor antigens in HCC make it an attractive target for immunotherapy. The principles of current immune-based therapies in cancer are harnessing the potential of the immune system to destroy malignant cells. Unfortunately, the existing immunotherapies for solid cancers rarely cure the disease once it has become metastatic.

**Figure 3:**
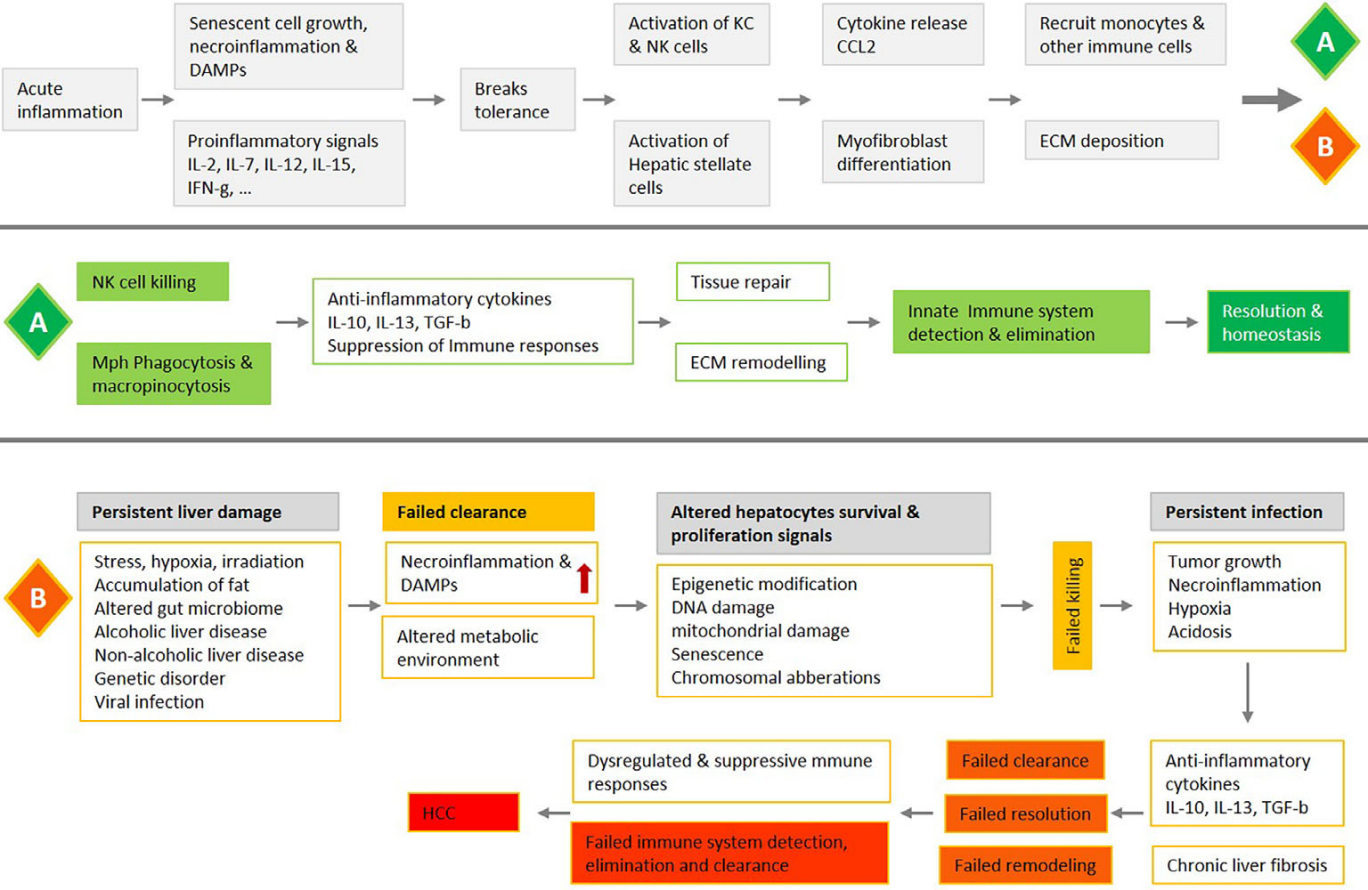
An overview of liver acute inflammation and two possible processes, resolution **(A)** or chronic inflammation and HCC **(B)**, with a focus on the NK-macrophage kill and clearance. Liver acute inflammation must be resolved for homeostasis to be stored. An array of diverse mediators regulates NK cells and monocytes/macrophages trafficking and blunt production of inflammatory mediators, while also promoting clearance of tissue. Failure in NK cells killing, and macrophage clearance are associated with chronic unresolved inflammation, which is the central causative factor in the development of HCC.

In our recent publication, we investigated the role of the necrotic zone and potential mechanisms involved in disarming macrophage phagocytic machinery in solid tumors (1). We suggested that further accumulation of dying, dead, and disintegrating cancer cells in this region makes the tumor proinflammatory and potentially provides a rich source of building blocks for the anaerobic metabolism of cancer stem cells as well as immune cells. The phagocytic clearance in the liver is fundamental for tissue homeostasis, controlling important aspects of inflammation and the immune response (**Figures 1**–**3**) (118). Herein, we discussed observations that support a synergistic act of NK-macrophage kill and clearance as a therapeutic strategy in HCC and other solid tumors. The key aspects of observations can be listed as follows:

- NK cells and macrophages have a primordial role in cancer immune surveillance.
- Synergistic act of kill and clearance by NK cells and macrophages is the centre point of innate and adaptive immunity, and therapeutic manipulation of this crosstalk provides an attractive strategy for HCC treatment.
- Excessive and persistent accumulation of necrotic and apoptotic corpses, debris, and senescent cells in TME support NK-macrophage kill and clearance strategy as a therapeutic tool in HCC (119, 120).
- NK cell deficiencies characterised by the absence of NK cells or their function and accumulation of TAMs with redundant phagocytic machinery indicate the importance of NK-macrophage kill and clearance is in tumor immune surveillance.

Further in-vitro and in-vivo experimental investigations could produce interesting findings that help to better understand the innate immune networking in HCC and increase therapeutic efficacy. Herein, our suggestive approaches to develop therapeutic methods based on NK-macrophage kill and clearance in solid tumors include: (i) The production of human-induced pluripotent stem cells derived NK cells and macrophages with the favorite functional features, (ii) chimeric antigen receptor (CAR) constructs of NK cells and macrophages that kill and clear efficiently, (iii) targeting the crosstalk between NK cells and macrophages, such as NKP30, to boost kill and clearance in HCC, (iv) immune checkpoint blockade, such as PD-L1/PD-1, and activation of cancer cell lysis and phagocytosis, (iv) blocking signaling axis like CCL2/CCR2 and CSF-1/CSF-1R to inhibit cell trafficking from bone marrow and TAMs accumulation in TME, (v) strategies to disrupting or inhibit HIF-1α and the related downstream genes to control metabolic switch in TME, (vi) and using nanorobots as a counterpart or substitute for immune cells to improve kill and clearance in TME (**Figures 2A–D** and **3**).

## Author Contributions

MM: conception and design of the manuscript, research of supporting evidence, writing and design of figures. FA and SM: editing, research of supporting evidence, critical revision of content, and final approval. All authors contributed to the article and approved the submitted version.

## Conflict of Interest

Author FA was employed by company Vitaccess.

The remaining authors declare that the research was conducted in the absence of any commercial or financial relationships that could be construed as a potential conflict of interest.

## Publisher’s Note

All claims expressed in this article are solely those of the authors and do not necessarily represent those of their affiliated organizations, or those of the publisher, the editors and the reviewers. Any product that may be evaluated in this article, or claim that may be made by its manufacturer, is not guaranteed or endorsed by the publisher.

## Abbreviations

BM, Bone Marrow; DAMPs, Damage-Associated Molecular Patterns; EMT, Epithelial-Mesenchymal Transition; GI tract, Gastrointestinal tract; HCC, Hepatocellular Carcinoma; HIF, Hypoxia-inducible Factor; HSCs, Hematopoietic Stem Cells; KC, Kupffer Cell; MDSC, Myeloid Derived Suppressor Cell (only monocytic); MHC-I, Histocompatibility Complex Class I; NK cell, Natural Killer cell; SDF-1, Stromal Cell-derived Factor-1; STAT, Signal Transducers and Activators of Transcription, TAM, Tumor Associated Macrophage; TCGA, The Cancer Genome Atlas; TME, Tumor Microenvironment; H&E, Hematoxylin-Eosin.
